# How Speakers Orient to the Notable Absence of Talk: A Conversation Analytic Perspective on Silence in Psychodynamic Therapy

**DOI:** 10.3389/fpsyg.2020.584927

**Published:** 2020-12-07

**Authors:** A. S. L. Knol, Tom Koole, Mattias Desmet, Stijn Vanheule, Mike Huiskes

**Affiliations:** ^1^Center for Language and Cognition Groningen, University of Groningen, Groningen, Netherlands; ^2^Department of Psychoanalysis and Clinical Consulting, Ghent University, Ghent, Belgium; ^3^Health Communication Research Unit, University of the Witwatersrand, Johannesburg, South Africa

**Keywords:** silence, conversation analysis, psychotherapy process research, psychodynamic therapy, single case study

## Abstract

Silence has gained a prominent role in the field of psychotherapy because of its potential to facilitate a plethora of therapeutically beneficial processes within patients’ inner dynamics. This study examined the phenomenon from a conversation analytical perspective in order to investigate how silence emerges as an interactional accomplishment and how it attains interactional meaning by the speakers’ adjacent turns. We restricted our attention to one particular sequential context in which a patient’s turn comes to a point of possible completion and receives a continuer by the therapist upon which a substantial silence follows. The data collection consisted of 74 instances of such post-continuer silences. The analysis revealed that silence (1) can retroactively become part of a topic closure sequence, (2) can become shaped as an intra-topic silence, and (3) can be explicitly characterized as an activity in itself that is relevant for the therapy in process. Only in this last case, the absence of talk is actually treated as disruptive to the ongoing talk. Although silence is often seen as a therapeutic instrument that can be implemented intentionally and purposefully, our analysis demonstrated how it is *co-constructed* by speakers and indexically obtains meaning by adjacent turns of talk. In the ensuing turns, silence indeed shows to facilitate access to the patient’s subjective experience at unconscious levels.

## Introduction

Psychotherapy is the incremental pursuit of exploring the patient’s past and its impact on the present. Session by session, the therapist and patient extend and build on matters discussed previously and in like manner, each therapy session is organized by sequences that produce topic development. Instead of an uninterrupted and ongoing exchange of turns of talk, however, therapy interaction also allows for extended moments of silence. If implemented skillfully, silences can encourage clients to reflect, to connect with their feelings and to continue with their line of thought (Hill et al., 2003). In concert, such silent moments give therapists room for observation of their clients and time to decide on how to respond and continue with the session, but also to refocus after distraction (Ladany et al., 2004). Followingly, silence is not (always) a sign of disengagement but, on the contrary, a multifunctional intervention type that possesses a communicative value albeit nonverbally (Lane et al., 2002; Ladany et al., 2004). The aim of the present paper is to examine how, from a conversation-analytic perspective, speakers in psychodynamic therapy^1^ orient to silences that occur in their interaction.

The approach of researching silence varies across disciplines. Psychotherapy process research (PPR) takes a more interpretive stance as it typically assesses phenomena such as silences by *post hoc* analysis and categorizes them according to the effect that speakers perceive and describe in retrospect (cf., e.g., Levitt, 2001; Hill et al., 2003). As such, PPR addresses the therapeutic benefits attributed to the use of silence and the various functions associated with it. Conversation analysis (CA), on the other hand, is concerned with the way speakers reach a point in talk where silence is a possibility and how they subsequently give meaning to this discontinuity in the following turns of talk (cf. Hoey, 2020). CA thereby provides a formal description of the sequential circumstances that result in the absence of talk and allows for detailed analysis of turns of talk that are adjacent to silences. In short, PPR assigns meaning to silence in retrospect based on individual interpretation, whereas CA describes how speakers collaboratively establish and continuously negotiate its meaning in the here and now.

Research into the perception and interpretation of silent moments in psychotherapy has shown that silence has the potential to facilitate a plethora of processes within each speaker’s inner dynamics. Such processes are described in the Pausing Inventory Categorization System (‘PICS,’ Levitt, 1998), which was assembled based on a grounded theory analysis of interpersonal process recall interviews. An interviewer replayed segments of the client’s last therapy session in which pauses of at least 3 s occurred. Clients were then asked to describe their experiences of these moments. This qualitative approach aimed at the exploration of clients’ experiences of pausing in psychotherapy in order to establish a manual that could be applied to therapy transcripts, also allowing for the examination of different types of pausing and their relationship with process measures (Levitt, 1998). Based on her grounded theory analysis, Levitt (1998) proposed a typology of silences that differentiates between disengaged, feeling, reflexive, expressive, associational and mnemonic pauses.

In a subsequent publication, Levitt (2001) divided these clusters into three highest order categories: productive or facilitative types of pausing, obstructions and neutral types of pausing. Congruent with what Ladany et al. (2004) would state in their later publication, Levitt (2001) stressed the heterogeneous character of the phenomenon due to the discrete categories she had identified. These authors thus ascribe the occurrence of silence to varied processes, while underlining that therapeutic silences are to be seen “as active moments instead of viewing them simply as moments in which discourse is absent” (Levitt, 2001, p. 306). Frankel et al. (2006) applied the PICS manual to data from client-centered psychotherapy sessions in order to assess good-outcome and poor-outcome therapies for the occurrence of productive, obstructive and neutral silences. Silences were selected according to the 3-s minimum criterion, then coded and their frequency compared to clients’ outcome scores. Their analysis suggests that therapists should stimulate silences if they appear to be emotional, reflective and expressive as these types of productive silences can be associated with good-outcome therapies (Frankel et al., 2006).

In the abovementioned studies, participants reflected on how they experience silences during therapy interaction through recall procedures and, as such, attributed meaning and function in retrospect. These qualitative reports provide valuable insight into the perception of silent moments during therapy sessions, into internal processes associated with them and how these relate to therapy outcome. The processes that underlie and result in silence were identified not because they were observable, but because participants were asked afterwards about their interpretation of these silences. At the opposite side of the spectrum, CA investigates what speakers actually do display when their talk is temporarily discontinued and as such takes a very different approach to the analysis of silence.

Conversation analysis is concerned with the dynamics of turns at talk between speakers, how they are locally managed and altogether sequentially construct interaction. The turn-taking model (see Sacks et al., 1974) provides a formal description of the sequential circumstances that are followed by an absence of talk. Turns of talk are allocated by a current to a specific next speaker, other speakers self-select or the current one continues talking. If these options are temporarily suspended by conversational partners, silence arises within a turn or in between turns. Apart from minor pauses or conversational gaps, CA treats silence as an interruption to the ongoing stream of talk or, in other words, as intervening “in the progressive realization of some interactional unit” (Hoey, 2020, p. 20). The positioning of silence thereby accounts for different types of silences. An absence of talk can occur as intra-turn pause or as inter-turn gap or as silence after a sequence-final turn (Sacks et al., 1974; Hoey, 2020). The latter type of silence is termed “lapse” and the focused-on silence in the current study. Lapses are defined as moments in talk at which all participants refrain from self-selection (Hoey, 2018).

Lapses can occur when speakers are engaged in ongoing alternative activities that require their focus and attention, which makes talk optional and silence allowable (Hoey, 2015). In the context of the present study, talk *is* the ongoing activity that both therapist and patient are engaged in. Hence, the occurring silences are not accounted for by other ongoing activities or alternative engagement. Hoey (2015) refers to such “silences where talk should be” (p. 442) as the conspicuous absence of talk and points out that a relatively static positioning of the participants’ bodies normally demonstrates that all parties are still committed to carry on with the conversational activity (based on data that was assembled in settings where participants were engaged in ongoing activities while talking). This is of course inevitably the case in therapy interaction, where therapist and patient remain in seated position, facing each other, until the end of session, and until then remain committed due to the formal contract of the session that both parties agreed upon.

From a conversation-analytic perspective, at least within Hoey’s research, silence thus accounts for a lack of progressivity in talk. As discussed in the above sections, psychotherapy process research takes a different stance and distinguishes between different types of silences when evaluating their impact on the interaction and, consequently, on the progressivity of the treatment itself. In the psychoanalytic approach to therapy, the phenomenon has gained an even more prominent role within the patient’s healing process. The observable level of talk, on which the phenomenon manifests, thereby gets surpassed and intrapsychic conflicts that let silence occur are taken into consideration as well. Psychoanalytic practice aims at elevating unconscious and repressed materials into conscious levels. The classical psychoanalytic view on silence was initially rather limited in that it interpreted silence as a form of resistance that undermines the analysand’s free association and thus production of signifiers (Gale and Sanchez, 2005). The analysand’s ego is thereby hold responsible for repressing the verbalization of unacceptable thoughts or feelings (Zeligs, 1961). According to Arlow (1961), prolonged silence therefore “demonstrates how unconscious resistance may be transformed into conscious reluctance to talk, and may be used very effectively to demonstrate to the patient the reality of a conflict which heretofore had been quite unconscious” (p. 50).

More recently, however, the Lacanian approach to talking therapy acknowledges the effect silence has on the chain of signifiers produced by the speakers as a form of punctuation (Pluth and Zeiher, 2019). Instead of a “lack of anything to talk about next” (Hoey, 2017, p. 129), Pluth and Zeiher (2019) characterize silence as a *rest* in the movement of language, which deliberately or undeliberately adds meaning to what has already been said. Therefore, silence is complementary to the signifiers produced by the speakers and not simply an absence of words (Sabbadini, 1991). Apart from its contribution to the meaning of words, it is also considered instrumental in developing the analysand’s ability to reflect, to internalize interpretations and in developing the capacity to be alone, which altogether “promote[s] the acquisition of insight” (p. 217, Gale and Sanchez, 2005). As such, silence is not conceptualized as an absence of therapeutic talk, but an inherent and meaningful part of therapeutic interaction. Silence can be used to facilitate, initiate or constitute specific therapeutic goals and is perceived as an integral part of the therapeutic toolkit.

In contrast, the current study examines therapeutic silence from a conversation analytical perspective (rather than as a therapeutic tool) and studies how silence emerges as an interactional accomplishment of the interactants within therapeutic discourse and how these silences attain their *interactional meaning* in and through the subsequent talk by participants. We will restrict our attention to one particular sequential context in which a turn by the patient comes to a point of possible completion (cf. Selting, 2000), followed by (or produced in overlap with) a continuer by the therapist upon which a silence of at least 3 s follows. In these cases the ensuing silence is an interactional accomplishment of both interactants as “non-talk (…) emerges when all participants demonstrably forgo the opportunity to speak at a TRP^2^, and persists until the production of some utterance that ends the silence” (Hoey, 2020, p. 30). The goal of this study is (1) to analyze these post-continuer silences with respect to their positioning within the larger episode of talk (where and when do they occur?), and (2) to examine how these silences indexically obtain interactional meaning by the adjacent utterances of the interactants. In our conclusion and discussion we will compare our analyses of the collaborative accomplishment of silence as an interactional practice to the manualized recommendations on silence as a therapeutic tool. We chose to conduct a single case study as we wanted to gain a complete and comprehensive view of the occurrence of silent moments within the larger course of the conversation at hand and in regard to the patient’s overall treatment trajectory.

## Materials and Methods

### Data

For this study, a single case was selected from the database of the Ghent Psychotherapy Study (GPS; Meganck et al., 2017), a randomized controlled trial on the treatment of major depression. With this single case design we investigate an individual patient’s treatment process (*N* = 1), i.e., intrasubject research (Hilliard, 1993). The use of the term “single case” is therefore distinct to the CA-coined idea of a single case in the form of an isolated manifestation of a particular phenomenon (cf. Sidnell, 2013). The case selection was conducted in the context of an overarching research project on the interactional practices of psychotherapy. We selected a case from the psychodynamic treatment condition of which recordings of all 20 sessions were available. We selected three sessions (1, 12 and 18) that were fairly evenly distributed across the treatment and therefore gave us a relatively complete overview of all stages of the therapy. These had been transcribed using the Jeffersonian notation (see Hepburn and Bolden, 2017). The data assembled within the GPS consisted of audio recordings, which limited our analysis to verbal communication. Therefore, visual aspects that come into play during silent moments, i.e., embodied behavior of the speakers, could not be included in the analysis.

### Participant Characteristics

The patient was a woman in her late fifties from Flanders, Belgium. As all patients in the GPS, she met DSM-V criteria of major depressive disorder. The patient further reported mild alcohol abuse that had been present for several months at the time of her intake. At the time of the treatment, she was single, divorced and in employment. In the years before, she had already sought counseling. The patient gave specific informed consent to let the audiotapes of the sessions being used for research purposes. The therapist was a 34-year-old male with 11 years of clinical experience. He had a postgraduate clinical training in psychoanalytic therapy. In order to conduct specific psychodynamic treatments for the purpose of the GPS, he had received additional training based on the Unified Psychodynamic Protocol for depression (UPP-depression; Leichsenring and Schauenburg, 2014). This protocol integrates empirically supported psychodynamic interventions for depression. In addition, the psychodynamic therapists that participated in the GPS were guided by Luborsky’s (1984) manual for psychoanalytically oriented therapy.

### Conversation Analysis

Eminently suited to study interactional phenomena, such as silences, is conversation analysis (CA), the methodological approach we chose to apply in this study. This inductive qualitative method aims at identifying the structure of language use – more specifically the practices of speaking and actions in talk that constitute that structure – based on the assumption that the speakers’ exchange and management of turns of talk persistently and unavoidably follows an orderliness or at least an orientation toward that orderliness (Maynard, 2013). With its sociological roots and close relation to ethnomethodology, CA research facilitates an unmotivated and mainly descriptive inquiry into the observable attributions and displays within participants’ conduct (Maynard, 2013). As such, it lends itself to be applied to all research contexts in which social interaction is at the center of interest. CA thereby follows robust research principles in that it uses meticulous transcriptions as research instrument (in addition to the original audio-recordings) and treats the conversational methods that speakers themselves display as evidence for its claims. In short, only what the participants make observable to each other is observable to the researcher and *only that* is thus reportable as evidence.

### Procedure

CA takes a relatively neutral approach in that it pursues an objective and unmotivated stance and focuses on local and situated procedures and achievements in talk while aiming at examining their generalizability across contexts (Svennevig and Skovholt, 2005). Its methodological procedure follows a systematic course of action: The data analysis starts with the examination of a single manifestation of a particular phenomenon (cf. Sidnell, 2013). After that, the researcher returns to the database in order to identify other excerpts in which the selected phenomenon occurs. The observations made during the initial qualitative analysis of the first case are then compared to and analyzed in light of the additional excerpts. At this point, the conversation analyst has assembled a collection of the researchable phenomenon and is as such working quantitatively in order to examine reoccurring conversational patterns or features of the phenomenon (cf. Maynard, 2013; Sidnell, 2013). The analysis of all excerpts still remains a qualitative inquiry – facilitated by complex transcription and the input the researcher receives from fellow conversation analysts during data sessions.

This study presents several excerpts that we find exemplary for the observations made during the data analysis. The advantage of transcription is that it preserves the spoken word, which would otherwise be as ephemeral to its investigator as it is for its speaker and receiver. Preservation makes it retrievable, examinable and representational of the actual event (Lapadat and Lindsay, 1999). A pause of x seconds as shown in the excerpts is thus merely a representation of the silence that actually occurred and only an observable fact because of the transcription. As such we, paradoxically, use transcription to capture a phenomenon that does not consist of a verbal event and is only represented by its measurement in duration.

Our initial criteria for the selection of excerpts concerned the sequential environment and the minimum duration of the silences. The initial analysis of excerpts in which extended moments of silence occurred, led to the identification of a particular sequential construction that we subsequently applied as selection criterion for assembling our collection. We selected excerpts in which a particular type of sequence construction manifested, consisting of a turn that receives a continuer and/or acknowledgment token prior to the silence. A total of 74 silences that manifested in this particular type of sequential environment were identified. Restricting our data collection to instances of post-continuer silence, provides control of the sequential environment (cf. Hoey, 2020). We applied the same 3-s minimum criterion as was applied in Levitt’s research (1998, 2001) and also in the studies that used the PICS manual for their data analysis (Frankel et al., 2006; Stringer et al., 2010; Daniel et al., 2018). The rationale behind the minimum duration of 3 s is that these silences are considered meaningful and not just accidental disfluencies (Stringer et al., 2010). This minimum pause duration thus appears as an accepted standard for research on silence. The sequences were constructed as follows:

• The patient’s turn (extended episode of talk or answer to one of the therapist’s questions)
• The therapist’s display of listenership (continuer)
• [Optional: The patient’s confirmation (acknowledgment token)]
• Silence of min. 3 s
• The therapist’s or patient’s next turn.

We chose to select excerpts in which the “mh mh” sound, a classic continuer, is produced prior to the silence (either adjacent to or in overlap with the turn by the patient). Hereby speakers explicitly forgo the opportunity to become the next speaker and demonstrate that their conversational partner is allowed to continue talking (cf. Gardner, 1998). Therefore the projected next action is the continuation of talk by the former speaker, i.e., the patient. The therapist thus abstains from claiming the next turn of talk and gives the patient the opportunity to further extend on the matter at hand. However, if both speakers forgo the opportunity to extend the current or to start a new turn, silence arises although the continuation of talk was projected by the use of the continuer. We therefore examined moments in the interaction where a notable absence of talk presents in order to analyze how speakers orient towards these silences.

## Results

In our data, 74 instances were identified in which silence manifested in post-continuer position. We found 17 instances of post-continuer silence in session 1, 34 in session 12 and 23 in session 18. In session 12 the patient presented with the highest degree of emotional distress. Therefore, the higher frequency in silences may have been due to the emotional processing during this session. Excerpt 1 gives an example of the particular sequential environment that, in this study, was used to identify post-continuer silences. The excerpt is taken from a larger episode of talk during which the patient reflects on the memories she has of her parents. Her relationship with her parents is a reoccurring topic in her treatment sessions and often elicits strong emotions. Our data revealed that the speakers often tended to accomplish silence during such emotional episodes of talk.

**TABLE 1:** Excerpt 1.

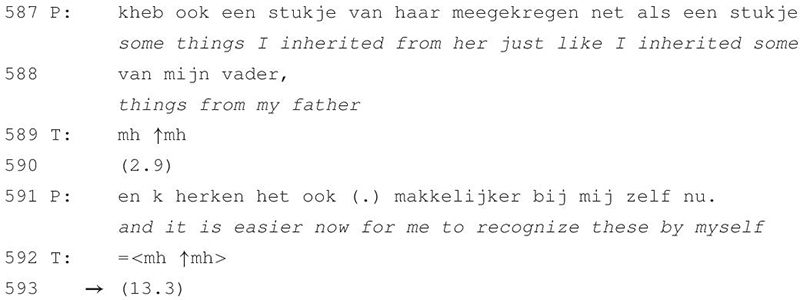

The excerpt is a prototypical example of the sequential context we investigated in this study. The patient’s line of talk reaches a point of possible completion as she states that she can see traits of her mother and father in herself (lines 587 and 588) and that she has now gained the ability to recognize these (line 591). At the first TRP, her turn receives a continuer from the therapist (line 589) and then again (line 592) after her turn-extension. A substantial silence of 13 s follows (line 593) during which both speakers forgo the opportunity to talk. The interactional meaning of such a silence is, however, indexically established by adjacent utterances and as such negotiated by the participants in the subsequent turns at talk.

In this study, we investigated the uptake after post-continuer silence, which led us to distinguish three distinct interactional environments based on the first turns subsequent to the silence, which speaker produces these and how they are constituted in relation to the ongoing discourse:

(I) After the silence the therapist produces a new turn in which he moves away from the subtopic discussed prior to the silence and returns to an overarching topic and/or agenda. In these instances the silence – *retroactively* – becomes part of a topic closure sequence. Silence thus marks the closing off of a “sequential environment where topic does not flow out of a prior topic” (Button and Casey, 1985, p. 4). With the following turn, a new (sub)topic gets nominated. This seems to be the prerogative of the therapist, as, in our corpus, we did not find examples of the patient redirecting the course of talk back to a former topic. When silence marks topical closure, there is no interactional orientation to the therapeutic function and/or meaning of the silence. We identified 27 of such instances in our data.
(II) After the silence the patient produces a new turn in which she elaborates on the topic discussed prior to the silence. This was the case in the majority of instances, namely in 45 out of the 74 excerpts. These elaborations are explicitly linked to the talk prior to the silence (using anaphora or other linking devices) and are often shaped as self-characterizations or as descriptions of emotional states. As such, the patient categorizes and/or summarizes the talk prior to the silence. In these instances the silence is interactionally – and again retroactively – shaped as an intra-topic silence. Although there is no explicit characterization of the silence as part of the therapeutic talk *per se*, the silence indexically is attributed interactional meaning by the adjacent utterances as part of a specific therapeutic activity (discussing/exploring topic X).
(III) After the silence the therapist’s next turn explicitly characterizes the silence as an activity and/or event. The turn contains a *formulation* (in the classical sense, cf. Garfinkel and Sacks, 1970) of the silence as a therapeutic event. This is quite rare as we only found two examples in our data, but it does show an explicit membership orientation to silence not as the absence of talk but as the presence of other therapeutically relevant events.

### Silence as Topical Closure

The first context we discuss where silence plays a role is in the negotiation of topic closure. With the initiation of the session, it is generally the patient who introduces a topic in response to an invitation by the therapist to tell about his or her current state of being. In the successive accomplishments of conversational projects, the speakers sometimes depart from the main topic in order to discuss, for example, additional background information or to reflect on emotions that were experienced at a particular point in time. Such conversational projects are the result of (sub)questions asked by the therapist that establish (sub)topics (van Kuppevelt, 1995). This sometimes leads to extended narrative episodes of the patient during which the therapist mostly demonstrates listenership, e.g., through the use of continuers, and is only sporadically claiming a turn. When the respective subtopic does not seem to elicit more material from the patient, the therapist formulates new interventions, such as requests for elaboration, or the subtopic gets closed off and the speakers move on to the next. This is similar to the structural organization found in cognitive and relational-systemic therapy sessions (cf. Bercelli et al., 2008). Also in psychodynamic therapy speakers alternate between inquiry and elaboration sequences. Silence, however, seems to play a role in the “closing off” of such conversational projects as can be seen in Excerpt 2.

**TABLE 2:** Excerpt 2 (session 1).

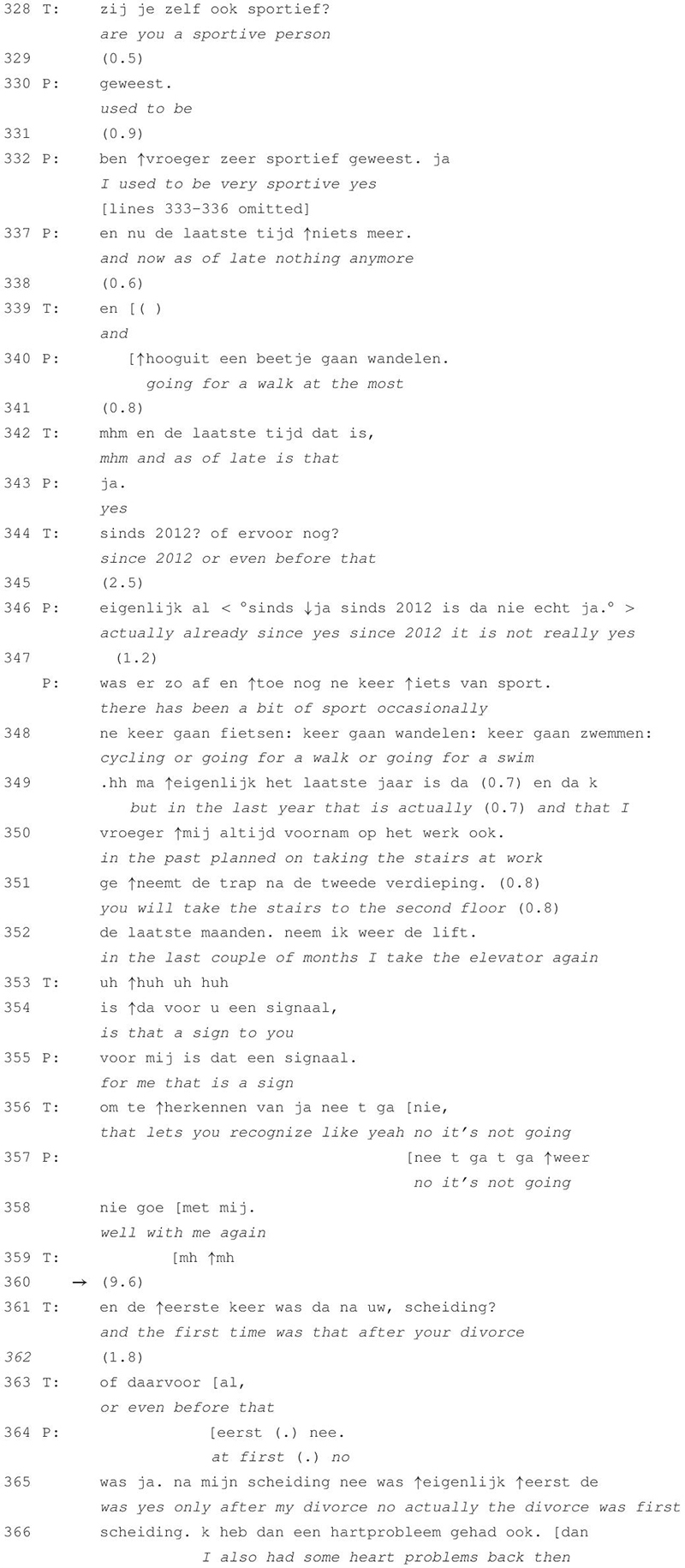

In this first session of the patient’s treatment, the speakers are establishing a timeline of the patient’s unstable wellbeing, discussing the first occurrence of depressive symptoms and how these were related to certain life events. Excerpt 2 shows an initiation of a subtopic by the therapist as he asks about the amount of exercise that the patient is doing and how this has evolved over the past years. This conversational project is situated along the sidelines of the session’s overarching topic, i.e., the review of the patient’s history of depression. After the patient’s extended telling ends and before moving on to the next inquiry sequence, both speakers remain silent for almost 10 s. The therapist’s next intervention then redirects the course of talk back to the overarching topic.

The therapist’s request for information in line 328 initiates a subtopic about the patient’s sporting activities. After summing up various types of sports that she did in the past (lines omitted), the patient concludes the list by mentioning that she is not participating in any type of sport lately (line 337) except for going for a walk occasionally (line 340). In response to the therapist asking for clarification on the point in time when she stopped doing sports (lines 342 and 344), the patient explains that the frequency of it has decreased since 2012 (lines 346–349). Since a couple of months she has even stopped taking the stairs at work and takes the elevator instead (lines 349–352). The therapist poses the question whether this is to be seen a sign (line 354), which receives confirmation in line 355. The therapist then extends the patient’s prior turn by adding “that it is not going well” (line 356), which gets in turn extended by the patient in lines 357 and 358 “no, it’s not going well with me again.” This turn receives a continuer by the therapist, which is, however, produced in overlap. After the silence of 9.6 s during which the patient’s turn does not get extended, the therapist initiates another inquiry sequence by asking whether she first experienced depressive symptoms before or after her divorce (lines 361 and 363), thereby redirecting the conversation back to the establishment of a chronological timeline. Through the use of the discourse marker “and” at the beginning of the turn, his request for information explicitly links back to the talk prior to the silence.

The intervention and response sequence prior to the silence is produced in the format of a collaborative turn-sequence (see Lerner, 2004) and connects the subtopic to the overarching topic by characterizing the amount of physical activity as an indicator of the patient’s state of mental wellbeing. In line 358 the sequence is potentially complete. The therapist’s use of the “mh mh”-sound in line 359 marks the receipt of the patient’s prior turn (Gardner, 1998). The continuer further demonstrates “the understanding that extended talk by another is going on by declining to produce a fuller turn in that position” (Schegloff, 1981, p. 81). During the silence in line 360, the patient is therefore given the opportunity to extend her turn and to further elaborate on the “signaling function” that taking the stairs has. As both speakers forgo the opportunity to talk and subsequently return to a prior topic, the silence here is constitutive of sequence as well as topical closure.

Silence thus appears to contribute toward the structural organization of talk as it retrospectively marks the closure of the sequence. With inquiry sequences therapists prepare the ground for elaboration and that elaboration is either accomplished by a series of therapist statements “grounded in previous clients’ talk” (Bercelli et al., 2008, p. 44) or, as we found in our data, by narrative episodes during which patients themselves further extend and elaborate on previous turns (as will be demonstrated in the analysis of Excerpts 3 and 4). The use of continuers in response to a patient’s turn demonstrates that the therapist treats this talk as potentially extendable (see Schegloff, 1981; Gardner, 1998) and leaves it up to the patient to make use of the opportunity for extension. In Excerpt 2, this opportunity remains unexploited. This results in an absence of talk after which the therapist initiates the continuation of the preceding topic. The speakers do not communicate about whatever caused or happened during the silence and as such, the discontinuity of the interaction is not treated as disruptive. Silence thus appears as an unproblematic and untopicalized occurrence that facilitates the transition to an alternative (sub)topic.

**TABLE 3:** Excerpt 3 (session 12).

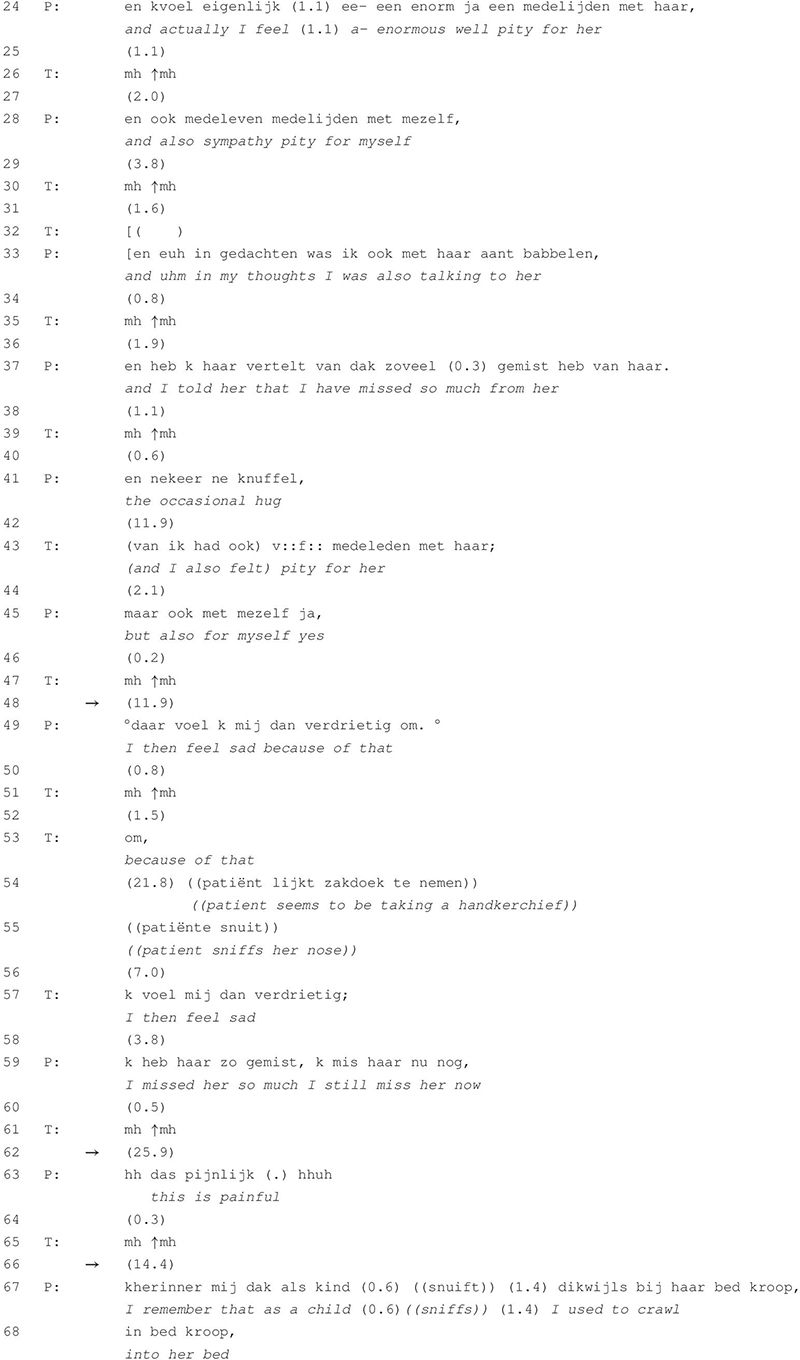

**TABLE 4:** Excerpt 4 (session 18).

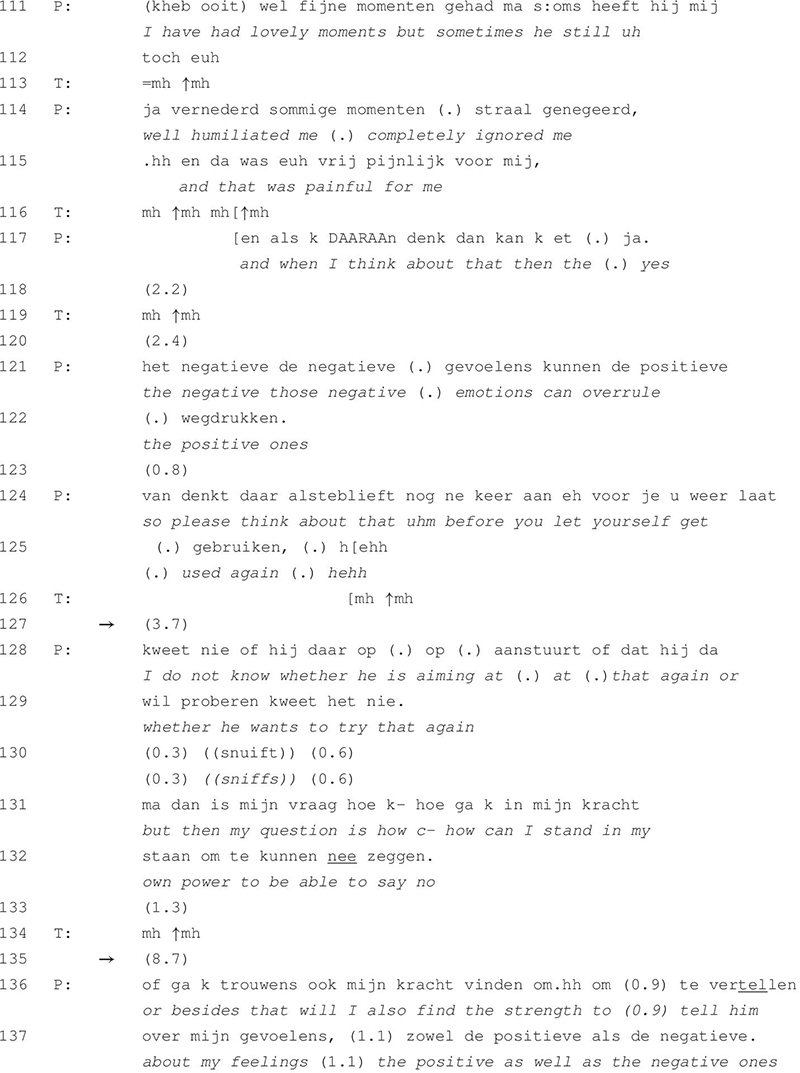

### Intra-topic Silence

Silence does not necessarily have to mark the closing off of previous sequences or subtopics. In other cases, speakers produce an extension of prior turns after extended moments of silence and as such, show an ongoing orientation toward the relevance of the topic at hand. However, these seconds during which both speakers remain silent are not treated as problematic or as a disturbance to the progressivity of the conversation. Instead, silence even seems facilitative of the patient’s insight as can be seen in Excerpts 3 and 4. In these excerpts, the patient continues talking after the silence by further elaborating on the respective topic. Although these “empty” seconds do not consist of talk, they accomplish the continuation of a line of thought that often results in more emotional statements by the patient.

In Excerpt 3 the patient is telling the therapist about her feelings of pity toward her mother and herself. At the beginning of the session she reported that she had cried that day because of the memories of her mother. In this succeeding episode of talk, she continuously extends her turns with short pauses in between while the therapist demonstrates listenership through the use of continuers and repetitions. The excerpt shows three post-continuer silences with a maximum of almost 26 s in line 62. Although the audio does not allow us to observe whether the patient is actually crying throughout this episode of talk, she notably gets emotional as is inferable from the production features: The turn in line 49 is produced with lowered voice volume, in line 54 she seems to be sniffing her nose, in line 63 the patient’s voice is wobbly and the turn is produced with aspiration at the beginning and a sigh at the end. These features display the patient’s distress and may indicate that there is crying (for a detailed analysis of different elements of crying see Hepburn, 2004).

The first post-continuer silence (line 48) occurs after the patient reports the huge feelings of pity that she experiences for her mother but also for herself as she has not received all the maternal attention that she needed (lines 24–41). The therapist then mirrors part of the patient’s prior turn (line 43) and uses the same personal pronoun as referent, thereby putting himself into the position of the patient. This form of repetition serves as an indirect request for elaboration (cf. Knol et al., 2020). Instead of further elaboration on the feelings of pity toward her mother, the patient treats the therapist’s other-repeat as a repair initiation and responds by repeating herself (“but also for myself, yes,” line 45). The therapist’s continuer in line 47 indicates that the patient is given the opportunity to further extend her turn. The ensuing post-continuer silence lasts for almost 12 s (line 48) until the patient states that she is feeling sad because of this (line 49). Another continuer is followed up with a single word-repetition by the therapist (“om,” a Dutch conjunction that can be glossed as “because of that,” line 53). The particle projects a subordinate clause to be completed by the patient and is thus functioning as an invitation to elaborate. It is produced with a slightly rising intonation contour, indicating stronger invitation for turn-extension than the preceding continuers. Instead of extending her telling, a silence of 22 s occurs (line 54). In line 57, the therapist again mirrors part of the patient’s preceding utterance, which invites continuation. The patient adds that she has missed her mother in the past and nowadays still continues missing her (line 59). This response once again receives again a continuer by the therapist after which both speakers remain silent for almost 26 s (line 62). In line 63, the patient states, with a slightly shivering voice and outbreath at the end of turn, that “this is painful.” The demonstrative links back to the utterances that precede the silence although it remains ambiguous where “this” refers to exactly (the memories of and feelings towards her mother or talking about them in the here and now).

Contrary to Excerpt 2 in which the silence is in retrospect indexical of topical closure, the silences in Excerpt 3 are implicitly treated as meaningful within the patient’s assimilation of emotions. This is established by the patient’s provision of an emotional interpretation, which is presented as a consequence of the thoughts and experiences that she reported prior to the silence. Excerpt 3 also shows that prolonged silences do not only occur as a single manifestation at a specific point in the interaction but that episodes of talk can contain multiple silences. The cumulative occurrence of these silences is not only a product of the patient’s slow pace in the production of turns but is also constructed by the therapist as he actively refrains from talking (except for selective repetition and the use of continuers). That he does not intervene but allows for these extended moments of silence to arise, demonstrates the consistent orientation toward encouraging the patient to independently continue elaborating. Here, silence leads to an extension of the (implicit) emotional content of the topic talk, which is enhanced by the therapist’s interventions. Silence thus seems to facilitate deeper insight into the patient’s emotions and inner conflicts, which the following excerpt is another example of.

Excerpt 4 is taken from session 18, which is relatively close towards the end of the patient’s treatment pathway. She reflects on her experiences with and related emotions for her ex-husband. At this point in treatment, the patient has already improved her ability to independently elaborate on the sources of her emotional distress and to come up with problem-solving lines of thought. In this episode of talk, she is contemplating whether to meet with her ex-husband and whether it is safe for her to talk about the negative feelings that past experiences with him have evoked. She is afraid that he may start emotionally abusing her again if she would give him the chance to. Similar to Excerpt 3, the duration of the between-speaker silences increases throughout this episode of talk with a maximum of almost 9 s in line 135.

The patient reports that she has had lovely moments together with her ex-husband (line 111) but immediately adds that at certain moments he had been ignoring her (lines 111–114), which caused a lot of pain (line 115). Whenever she is now thinking back, the negative emotions overrule the positive ones (lines 117–122). In lines 124 and 125, she formulates an imperative turn construction as if she is reminding herself of these negative memories in order to prevent getting abused. The therapist produces continuers throughout the patient’s telling (lines 113, 116, 119, and 126). After a lapse of almost 4 s, the patient shares her doubts about her ex-husband’s intentions (lines 128 and 129). In lines 131 and 132, however, she redirects the focus of her talk back toward herself by declaring that she is looking for a way to say no. This turn again receives a continuer by the therapist after which both speakers remain silent for 8.7 s (line 135). In her succeeding turn, the patient remains reflective about her own ability, asking herself whether she will find the strength to tell her ex-husband about her feelings (lines 136–137).

Excerpts 3 and 4 show that silence is facilitative of reflection. The therapist does not close off the sequence after the occurrence of silence but encourages elaboration by producing turns that mirror the patient’s preceding utterances or through the use of continuers. As such, he seems to remain in the backseat of the conversation, providing support and encouragement for the patient’s independent elaboration on the respective topic without being too directive in his interventions. In Excerpt 3, this results in an evolution of the patient’s talk from a descriptive stance toward an emotionally reflective one. Excerpt 4 shows a similar evolution in that the patient’s talk moves from the description of negative and painful memories toward a more proactive stance that is reflective of her own competencies in dealing with the (emotions for her) ex-husband.

### Silence as a Therapeutic Event

In our data, the participants rarely reflected on the silence itself. Whenever this was the case, silences were notably longer in duration. The following excerpt presents a moment in the therapy session during which a remarkably long silence occurs that seems facilitative of the patient’s exploration of feelings that she is experiencing (line 769). In contrast to the two preceding examples, however, this does not manifest implicitly in the patient’s succeeding talk but is explicitly pointed out by the therapist. In the intervention that succeeds the silence, the therapist topicalizes this disruption to the ongoing talk (line 770). In her response in line 772, the patient reports on the cause of this disruption as having a peaceful feeling.

Excerpt 5 shows two episodes of talk. In lines 648–672 the participants arrive at a turning point within the session as the patient discovers that her parents’ attributes and behaviors not necessarily have to be transferred onto the next, i.e., her own, generation. She states that this revelation gives her the feeling of “letting go” (line 662). The therapist adds that she is now finally able to break free (line 664), however, “not in a rush” (line 668) but, as the patient extends his turn, by “gently letting go” (line 672). After this collaborative turn-construction (see Lerner, 2004), the participants elaborate on the peaceful feeling and the support she experiences from the people surrounding her (lines omitted). The patient points out the she has come to the revelation because of the inner conversation she had with herself, which was possible because she had the day off. She elaborates on her work schedule, after which the therapist brings in a positive assessment about the type of work that the patient is doing (lines 757 and 759). After stating that she still finds it joyful to continue working at a restaurant, the conversation arrives at a notably extended silence of almost 72 s (line 769). The therapist then topicalizes the silence in line 770 by guessing that she was lost in thought during the silence. The patient responds that she experiences a peaceful feeling (line 772). After another but shorter silence of 8.5 s (line 775), the therapist asks whether she is feeling sleepy (lines 776 and 780) to which the patient responds that she is actually feeling energetic (lines 782 and 783).

**TABLE 5:** Excerpt 5 (session 12).

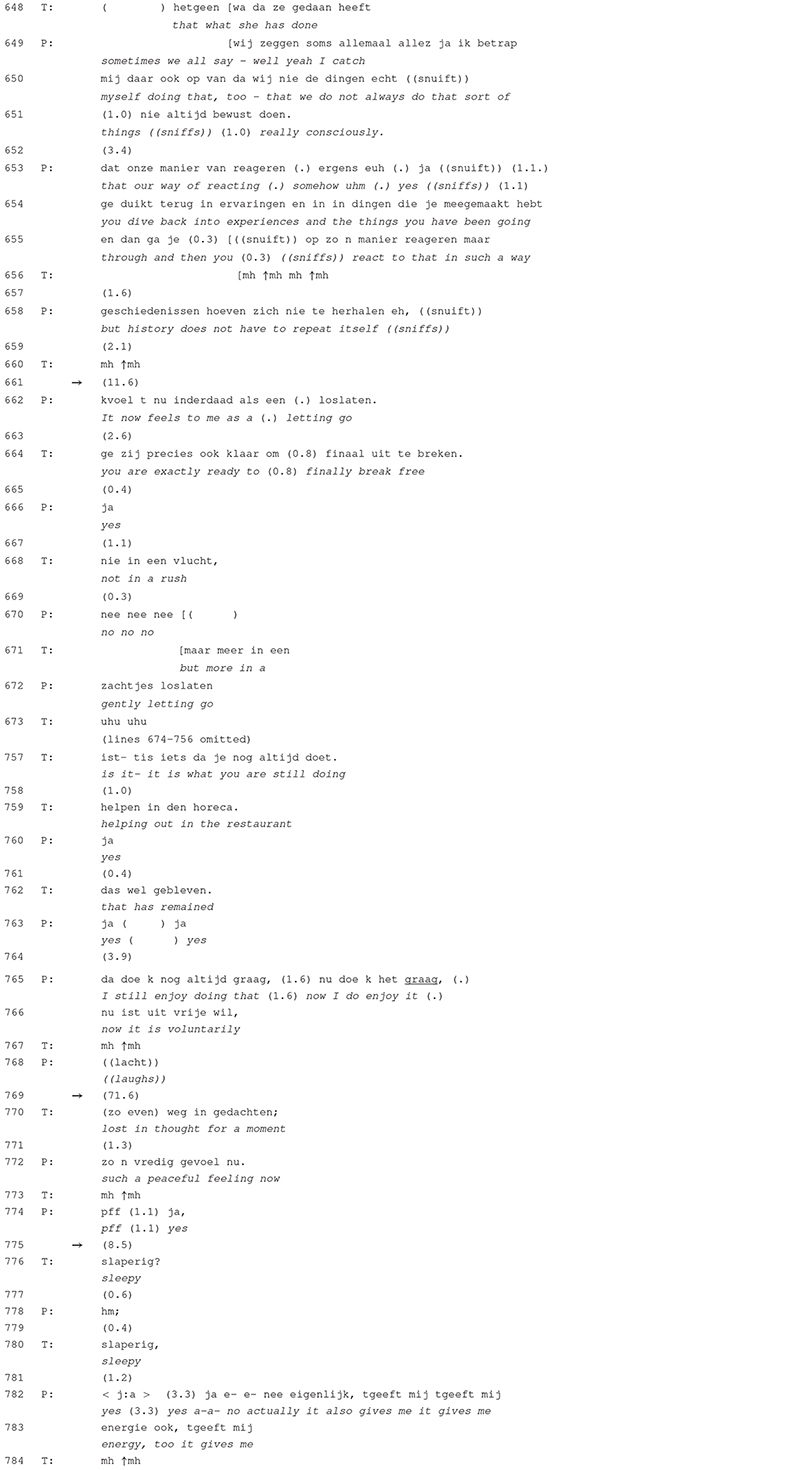

In this excerpt, the silence is treated differently to the silences in Excerpts 2–4 as it becomes topicalized in the form of a meta-communicative description and as such brought to the surface of the conversation. More specifically, it is not the silence itself that is topicalized but the activity that took place during the silence (being lost in thought). In the other examples, moments of silence are not treated as disruptive but simply become integrated into the course of talk. These silences were, however, notably shorter as they only reached a maximum duration of 26 s. In Excerpt 5, the disruption is made explicit because of the therapist’s guess on what caused the silence. The question remains whether this may also be related to its duration. Both speakers remain silent for more than a minute, which indicates a more substantial discontinuity in the discourse than in the other instances. Although the progressivity of talk is temporarily put on hold, the speakers do not treat this in a negative sense. The patient assigns a positive feeling as the cause (the peaceful feeling) while the therapist seems to be keen on assigning a more psychophysiological cause (feeling sleepy). Whenever such underlying processes become the topic of the conversation, as in Excerpt 5, participants show an orientation toward assigning a function to the silence. Such instances can be analyzed by means of CA but also allow for an interpretive analysis as it is common in PPR.

This analysis demonstrated that after inquiry sequences, further elaboration by the patient as projected by the therapist’s use of continuers, is not always produced, which results in the occurrence of silence between the speakers. After that, either the therapist launches an elaboration sequence through the use of mirroring and/or follow-up questions or the patient continues talking and if so, these utterances are produced with a more emotional stance. Silences appear cumulatively and not as single manifestations, and throughout such episodes of talk the between-speaker silences tend to increase in duration. Occasionally, prolonged silences become topicalized and only in these cases, the potential disruption of talk is actually treated as disruptive.

## Discussion

Irrespective of the type of interaction, sequence endings “systematically provide for the occurrence of silence” (Hoey, 2020, p. 11). In the context of psychotherapy, the therapist can opt to intervene whenever patients fall silent in order to maintain the progressivity of talk. Especially in the psychoanalytic tradition, however, silence has been recognized as a meaningful contributor to the therapeutic relationship and valuable in assisting the patient to connect with his or her subjective experience at unconscious levels (Warin, 2007). According to Sabbadini (1991), silence can “transform unconscious anxiety, concerning some as yet unknown and unworked-through inner conflict, into more manageable, though often more painful, conscious anxiety” (p. 409). Excerpt 3 showed an example in which the patient, after an extended period of silence, reported on painful emotions evoked by the memories of her mother. The silence here thus seemed to have facilitated access to deeper layers of the patient’s repressed memories. These findings are thus consistent with the aforementioned functions attributed to silence and its value for the therapeutic process (cf., e.g., Gale and Sanchez, 2005).

One aspect that contrasts the psychoanalytic discourse to regular social interaction is that the analyst allows the analysand to speak at great length, only interrupting him or her if that is absolutely necessary for the analyst’s understanding, thereby approaching the analysand from his or her own frame of reference as much as possible (Fink, 2007). When silence occurs in the analysand’s discourse, it provides “a gateway that leads from the conscious to the unconscious and can be used to enhance and enable self-exploration” (Warin, 2007, p. 48f.). Still, in Excerpts 2 and 5, the therapist himself eventually chose to intervene by producing a next turn of talk. The research method that was used in the current study enabled us to investigate *how* the therapist constructed his subsequent turns. CA, however, does not provide the instruments to identify what determined the therapist’s decision to break these silences. In Excerpt 2, the therapist redirected the talk back to the overarching topic without addressing the silence or its underlying processes. Maybe he had the impression that the silence would not elicit more fruitful material or maybe he felt as if the patient was struggling too much with inner conflicts. Another possible explanation may concern the phase of treatment as this example was selected from the first session and the therapist may have prioritized the review of the patient’s history over possible introspection at that point. Interestingly, in our data, it was always the therapist who initiated topical closure after silence (by initiating new or returning to prior topics). Hence, regarding topic management, this asymmetry points toward a division of roles, with the therapist being the one who is more inclined to guard the topical structure of the interaction.

The duration of the silence in the last example in our analysis (Excerpt 5), was remarkably long in comparison to the other silences that were presented. Another striking aspect of that episode of talk was that after the revelation and the patient’s description of her feelings as “breaking free,” the speakers had already moved on to a more neutral topic when the silence occurred. The effect of the positive emotions she described was possibly delayed as she fell silent during the more general elaboration on her employment in the restaurant. Holding a silence for a long time thereby allows for visualizations to become brighter and emotions clearer (cf. Warin, 2007), which would be a possible explanation for her response to the therapist’s question that broke the silence. According to Sabbadini (1991), prolonged duration of a silence also makes it increasingly harder to break it. This may account for the topicalization of the silence as it possibly was “the elephant in the room” and probably safer to address than to formulate an intervention that aims at continuing with what came prior to the silence.

The four examples further illustrated that although it is primarily after a patient’s turn that the speakers fall silent, it is also the therapist who temporarily forgoes the opportunity to talk. Until one of the speakers self-selects and continues speaking, the silence thus exists as an interactional product of both speakers’ verbal disengagement. This also supports the assumption that “therapists are more active participants of a communicative ‘system’ than traditional psychoanalytic theory would assume” (Buchholz, 2019, p. 814). Hence, as Sabbadini (1991) states, silence is an interpersonal phenomenon that “can take place only within a relationship” (p. 410). What remains unaddressed in the psychoanalytic literature on silence, however, is how it relates to therapists’ use of continuers, such as the “hmm”-sound. This form of recipient talk is most frequently used in psychoanalytic practice, as this technique expresses attentiveness and encourages the analysand to continue talking while the meaning of this sound remains difficult for the analysand to uncover (Fink, 2007). Delaying a response in such a way helps patients “to develop their troubles stance in more detail” (Muntigl et al., 2014, p. 33). Our analysis supports this finding since, in the case of intra-topic silence, the use of continuers and the ensuing silences seemed to elicit further emotive elaboration by the patient. The absence of talk provided for a moment to evaluate what had been said and to then slightly move away from it in order to articulate a somewhat greater understanding or result that the prior talk has built up toward.

Previous research on the meaning and function of silence in the context of psychotherapy attributes a significant value to its use (cf., e.g., Gale and Sanchez, 2005; Frankel et al., 2006). On the one hand, it can be implemented intentionally as a therapeutic instrument, serving various beneficial purposes within the therapeutic process if sensitively employed by the therapist (Lane et al., 2002; Ladany et al., 2004). On the other hand, the ability to remain silent in the presence of someone else is also understood as “an achievement on the part of the patient” (Winnicott, 1958, p. 29). And even from a conversation analytical perspective, “the developing of an ability in a relationship to be silent” is acknowledged as a crucial aspect of interpersonal interaction (Sacks, 1995, p. 50). Our study is complementary to these findings. We demonstrated that silence is constituted as an interactional achievement by both speakers and that its meaning (on an interactional level) is indexically established in the speakers’ adjacent turns of talk. Our analysis showed that if silence becomes part of a topic closure sequence, speakers do not show an interactional orientation to the therapeutic function or meaning of the silence. If silence is co-constructed by the speakers in the form of an intra-topic silence, speakers implicitly attribute interactional meaning to it within the subsequent utterances. These silences are constituted in the ongoing discourse as moments that allow continuing with the current course of action in order to reach a therapeutically relevant point. As such, silence becomes part of the therapeutic activity at hand. Lastly, if silence between the speakers explicitly gets topicalized and therefore oriented to as an event in itself, it is not referred to as an absence of talk but as the presence of other therapeutically relevant processes and is treated as a resource for building a next action.

The current study was limited to the occurrence of silence in a specific sequential environment, namely a sequence construction in which the therapist’s use of continuers prior to the silence projected the continuation of the patient’s current stretch of talk. This continuation was either accomplished after a moment of silence or the patient did not extend her turn at all. Our analysis, however, showed that, in most cases, speakers in psychodynamic therapy do not treat the discontinuity of talk as disruptive. The therapist switched topics or formulated interventions that elicited further elaboration. He did so without using hesitation markers or other markers of reluctance that would display an awareness of the unaccomplished projection of the use of continuers. When the patient extended her turn after the silence, she provided material that demonstrated a more emotional stance as if the absence of talk had facilitated access to deeper levels of her unconsciousness. Whenever the silence was actually treated as a discontinuity of talk, speakers made this explicit by referring to the underlying processes that manifested during the silence. Followingly, the meaning that an absence of talk receives within the respective episode of talk, depends on its relation with the adjacent turns (cf. Hoey, 2015, 2020).

Our findings suggest that silence bears a potential to become interactionally – and therefore also therapeutically – meaningful, but that it is up to the speakers to make use of this potential. Using conversation analysis, we provided a detailed picture of the speakers’ practices of speaking leading up to silence and how they can make the occurrence of silence therapeutically relevant in their subsequent turns of talk. Although the phenomenon had already received a fair amount of attention in psychotherapy process research and in psychoanalytic theory, this study shows how CA research can contribute to the practitioner’s knowledge base, such as treatment manuals and therapy training. Investigating psychotherapy through the lens of CA provides extensive and microdetailed descriptions of actual interactions and thereby opens new dimensions to the existing understanding of therapy as a practice (for an extensive report on the contribution of CA findings to the stocks of interactional knowledge, see Peräkylä and Vehviläinen, 2003). Empirical research into social interaction thereby shows how professional knowledge, i.e., theoretical concepts and ideas, become translated by participants into situated conversational behavior. Silence in psychotherapy seems to be the golden ticket that gives access to insight and emotional awareness on the part of the patient. CA research, such as the current one, informs practitioners in more concrete terms about the sequential environments in which silence occurs, about the observable features of these environments and, consequently, how participants deal with silence as an interactional resource.

## Author’s Note

The data for this research was collected as part of the Ghent Psychotherapy Study (Meganck et al., 2017), a randomized controlled trial on the treatment of major depression (registered on the Open Science Framework; ISRCTN 17130982). Our sample consisted of a small subsample of audio-recordings and was selected without consideration of outcome measures.

## Data Availability Statement

The data collection generated for this study consists of anonymised transcript excerpts and is available on request to the corresponding author. Requests to access these datasets should be directed to a.s.l.knol@rug.nl.

## Ethics Statement

The Ghent Psychotherapy Study was approved by the Ethical Committee of the University Hospital of Ghent University. All participants provided their written consent to participate in this study.

## Author Contributions

AK: main author of the manuscript, main investigator responsible for data-analysis and interpretation. TK: reviewer of the manuscript, contributed to the study design and to the data-analysis and interpretation. MD: reviewer of manuscript, contributed to the to the study design and to the data-analysis and interpretation. SV: reviewer of the manuscript. MH: main reviewer of the manuscript, contributed to the study design, performed the data-analysis, and contributed to the interpretation of the results. All authors contributed to the article and approved the submitted version.

## Conflict of Interest

The authors declare that the research was conducted in the absence of any commercial or financial relationships that could be construed as a potential conflict of interest.

## Footnotes

1 The single case investigated in this study was selected from the psychodynamic treatment condition of the Ghent Psychotherapy Study (GPS). The therapist followed psychodynamic treatment protocols for the purpose of the GPS but had been trained in psychoanalytic therapy. In this paper, the term psychodynamic therapy is therefore used synonymously to the term psychoanalytic therapy.

2 Transition Relevance Place (TRP) refers to points of possible completion “which make turn transition relevant but not necessary” (Selting, 2000, p. 478).
